# Sensor and Video: Two Complementary Approaches for Evaluation of Dairy Cow Behavior after Calving Sensor Attachment

**DOI:** 10.3390/ani11071917

**Published:** 2021-06-28

**Authors:** Johanna Pfeiffer, Olivia Spykman, Markus Gandorfer

**Affiliations:** 1 Bavarian State Research Center for Agriculture, Institute for Agricultural Engineering and Animal Husbandry, 94099 Ruhstorf an der Rott, Germany; olivia.spykman@lfl.bayern.de (O.S.); markus.gandorfer@lfl.bayern.de (M.G.); 2 TUM School of Life Sciences, Technical University of Munich, 85354 Freising, Germany

**Keywords:** activity, Brown–Forsythe test, digital, variance, video

## Abstract

**Simple Summary:**

We analyzed whether attaching sensors to the tail of cows for the early detection of calving leads to behavioral changes. In this study, we combined conventional video analysis with data from digital sensor technologies. To detect the potential agitation of cows after calving sensor attachment, we analyzed cow activity behavior. Based on the combined results of video and sensor analysis, there was no clear evidence that attaching sensors to the tail generally altered the ethological pattern of all cows analyzed. However, the investigation of individual cows showed an increase in the frequency of tail raising and rubbing the tail after calving sensor attachment. These changes would be worth analyzing in more detail on a larger scale.

**Abstract:**

Studies evaluating calving sensors provided evidence that attaching the sensor to the tail may lead to changes in the cows’ behavior. Two different calving sensors were attached to 18 cows, all of which were equipped with a rumen bolus to record their activity. Two methodological approaches were applied to detect potential behavioral changes: analysis of homogeneity of variance in cow activity (5 days pre-sensor and 24 h post-sensor) and analysis of video-recorded behavior (12 h pre- and post-sensor, respectively) in a subgroup. The average results across the sample showed no significant changes in the variability of activity and no statistically significant mean differences in most visually analyzed behaviors, namely walking, eating, drinking, social interaction, tail raising, rubbing the tail, and the number of standing and lying bouts after calving sensor attachment. In addition to considering mean values across all cows, individual cow investigations revealed an increased number of time slots showing a significant increase in the variability of activity and an increased frequency of tail raising and rubbing the tail on objects after calving sensor attachment in some cows, which should be investigated in more detail on a larger scale.

## 1. Introduction

### 1.1. Sensor Systems to Improve Calving Management

Calving monitoring and assistance can reduce the incidence of stillbirths and the proportion of cows with post-partum endometritis and uterine infections while also having a positive effect on reproductive performance [1,2]. To improve calving management, sensors for early calving detection are discussed as a technological solution. These sensors can detect parameters such as behavior or body temperature, which change a few hours before calving [3]. Saint-Dizier and Chastant-Maillard [3] described four different types of commercially available calving detection devices: accelerometers and inclinometers that are attached to the tail and measure activity, abdominal belts to monitor uterine contractions, vaginal probes to monitor vaginal temperature, and devices in the vagina or on the labia that detect expulsion of the calf. Predicting the onset of calving through the continuous monitoring of tail movement is possible due to changes in the behavior of a cow before calving, such as a significant increase in the frequency and duration of tail raising [4,5]. There are currently three different market available sensors for attachment to the tail, which differ in the way they are attached: with a ratchet (Moocall, Moocall Ltd., Dublin, Ireland), with an adhesive and tape (CalveSense, Allflex Group Germany GmbH, Bad Bentheim, Germany), and with a clamp and tape (Calving Alert Set, Patura KG, Laudenbach, Germany). In addition, many sensor systems that were originally used exclusively for estrus detection have been further developed in recent years to include the function of calving monitoring. Parameters such as activity, temperature, or rumination are used for this purpose. The available literature shows that some sensors on the market have the potential to achieve satisfying results in the early detection of calving [5,6,7,8,9]. Studies on tail-attached sensors have reported sensitivities of up to 95% [5].

Although the literature suggests that sensors can help to improve calving management and, therefore, specific animal welfare aspects (e.g., calf mortality), there is also evidence that sensors attached to the cow’s tail in particular can cause behavioral changes or even injuries. Studies evaluating tail-attached calving sensors have so far focused on the Moocall sensor. Investigations on the behavior of dairy cows after attaching a calving sensor to the tail are, however, rare. In this context, Lind and Lindahl [10] reported on practical experience with the Moocall calving sensor. Their study on cow behavior after the attachment of the sensor system had to be discontinued due to frequent tail injuries. They subsequently conducted telephone interviews with 15 farmers and confirmed these initial experiences: 80% of the farmers stated that the behavior of the cow worsened after the Moocall sensor system had been attached, which they inferred from increased tail raising and fidgeting. Additionally, 87% noticed injuries to the tail of the animals. However, the interviewed farmers’ assessment that the cows’ behavior changed in a way that was judged to be negative after attaching a Moocall sensor [10] was a visual and subjective one, as it was not recorded according to a uniform classification scheme. Recently, Giaretta et al. [5] investigated precision of calving prediction with the Moocall sensor and dairy cow behavior after its attachment. They visually analyzed walking, eating, lying down, standing, and tail movement one day before and on the day of calving sensor attachment, concluding that the Moocall sensor was well tolerated by the cows, as only eating behavior showed an increase after its attachment to the tail. Voss et al. [8] conducted a study on the Moocall sensor, focusing on its fit on the tail and skin integrity after attachment. In their experiment, the Moocall sensor did not continuously remain on the tail in 86.1% of the animals, with reason being mainly categorized as ‘fell off tail’ or ‘tail swollen or painful’. 

### 1.2. How Can Behavioral Changes in Dairy Cows Equipped with Tail-Attached Calving Sensors Be Assessed?

To assess behavioral changes or even stress in cows, various parameters have been analyzed and different approaches have been applied in literature. The extent to which animals respond to stress depends not only on the duration and intensity of exposure to a stressor, but also on environmental conditions, physiological status, and previous experience with the stressor [2]. So far, the analyzed stressors for cows include isolation from the herd [11,12,13,14], confrontation with novelty such as a milking system [15,16,17], exposure to stray voltage [18,19,20,21], or heat [22,23,24,25,26,27,28,29,30,31], among others. The previous studies showed that cows responded to stress by increased activity [12,14,16,18,20].

To describe changes in the behavior of an animal, the mean and median of activity data from different time periods are often used [20,29,32]. In addition, analyzing variability of activity and movement behavior is a common methodological approach to characterize and predict, among others, diseases in cows [33,34,35,36,37,38]. For instance, Edwards and Tozer [33] showed a reduced average walking activity, recorded with pedometers, in cows with metabolic or digestive disorders compared to healthy cows. Their study also indicated that the variability of activity was higher in sick cows compared to healthy ones, regardless of lactation number and calving season. Thus, multiple studies indicate that not only the activity level per se is relevant for describing cow behavior, but rather it appears that the scattering of activity values around a mean value can provide further valuable information.

Digital sensor technologies for dairy farming are currently being used with the argument of improving animal welfare as they contribute to calving and health management. At the same time, there are concerns as to whether the attachment of sensors has negative effects on cows. In the worst case, there could be a contradiction: Does a digital technology with the intention to improve animal welfare by monitoring calving actually disturb cows? This study therefore addresses the question of whether the attachment of two different calving sensors leads to behavioral changes indicating that cows are being disturbed by these monitoring devices.

## 2. Materials and Methods

### 2.1. Data Collection

#### 2.1.1. Animals and Housing

The data (mid-July to early December 2018) originate from a dairy research and demonstration farm located in Bavaria, southern Germany. On the dairy farm, cows were kept in a free-stall barn year-round. Six to eight weeks before the expected calving date, the animals were dried off and moved to the dry cow area. Approximately eight days before the expected calving date, the cows were separated into one of four maternity pens littered with straw. The animals were paired in the maternity pens solely dependent on their expected date of calving and thus independent of age, lactation, breed, and type of calving sensor attached. In the maternity pen, the total mixed ration was provided daily at the same time (09:15 to 09:30 a.m.) and water was available ad libitum. Two video cameras were placed above the four maternity pens and recorded continuously. Mobotix D15 cameras were used, which ensured good night vision by means of an integrated infrared lens. The video recordings were stored in a network-attached storage. The analysis included 15 cows and 3 heifers (mean age ± SD = 5.5 ± 2.6 years; mean parity ± SD = 4.1 ± 2.5), of which eleven were Simmentals, four were Brown Swiss, and three were Holstein breed (see Table A1 in Appendix A).

#### 2.1.2. Sensors and Calving Management

On this dairy farm, cows were equipped with a calving sensor and received a rumen bolus (smaXtec animal care GmbH, Graz, Austria) several weeks before the expected calving date. We used Moocall (Moocall Ltd., Dublin, Ireland; weight: 329 g including pad) and CalveSense (Allflex Group Germany GmbH, Bad Bentheim, Germany; weight: 172 g) as sensors for an early detection of calving. Both calving sensors analyze tail movement and issue a message to the farmer a few hours before calving. Therefore, they are attached to the cow’s tail only a few days before the estimated calving date. As tail-attached calving sensors are only applied for a short period before calving, the behavioral analysis focused on a short-term adaptation period (see also [5]). The user has no insight into the recorded raw data from the calving sensors, but only receives messages in the case of an imminent calving. As it was not the purpose of this study to analyze the calving alarms of the sensor systems themselves, this was not a limitation. The second type of sensor, a rumen bolus, is a commercial product administered orally into the reticulorumen using an oral applicator. In the reticulorumen, it continuously records animals’ activity in ten-minute intervals on a scale between 1 and 100 as a dimensionless index using a 3D acceleration sensor. It additionally measures core body temperature with an accuracy of ±0.05 °C [39]. The data is then sent to a base station, from where it may be exported for analysis. The activity measurement is not influenced by rumen motility since disturbance factors that cannot be attributed to the movement of the animal itself (e.g., movement of the rumen) are filtered out [40]. The smaXtec bolus has already been applied in scientific studies to clarify a range of research questions (see [41,42,43]). Studies on the performance of the activity-based estrus detection of the smaXtec bolus showed a sensitivity of 92% (blood progesterone as reference) and a precision (positive predictive value) of 89% [44]. Based on a review of estrus detection rates in other sensor systems [45], this accuracy can be classified as good.

In the maternity pen, either a Moocall or CalveSense sensor was attached to the tail five days in advance of the estimated calving date. The attachment of the calving sensors was carried out by only two staff members, who received instructions and training on the correct handling of the two sensor systems with cows outside the study cohort before the start of documentation. As such, a learning process of attaching the sensors, which could potentially influence the animals’ behavior could be excluded. Sensor attachment was performed according to the respective manufacturer’s guideline: the Moocall sensor was fastened with a ratchet whereas the CalveSense sensor was secured to the tail with an adhesive and tape. After the calving sensor attachment, the farm staff checked its fit on the cow’s tail and for potential pressure marks or swellings on the tail. The calving sensor check was performed one to two hours after its attachment and then twice daily during the routine inspection of animals to calve. In this process, any abnormalities such as swelling of the tail, conspicuous behavior such as rubbing the tail on objects, or sensor dropping after attachment were documented. As recommended by the manufacturer, Moocall sensors were removed for three to four hours if they had already been attached to the tail for four days (and the cow had not yet calved) before being reattached.

On the dairy research and demonstration farm, CalveSense sensors were attached to a total of 36 animals and Moocall sensors were attached to a total of 37 animals. Due to the defined period of analysis and not all the calving sensors attached remaining on the tail (e.g., due to falling off), 18 animals could be included in methodological approach 1, and nine of them also in methodological approach 2. The analysis included only animals on which a calving sensor remained attached to the tail for at least 24 h (exception: cow 15 with sensor being removed after 23 h and 15 min). 

Because a cow’s behavior may be influenced by its overall state of health [46,47], we also considered health data for the analysis. As the dairy farm is participating in ProGesund, an information service for veterinarians and farmers, documentation on all diseases diagnosed by a veterinarian was available. Prior to calving sensor attachment, a health assessment of the animals was performed, which included a visual assessment of the animal as well as continuous data on core body temperature and number of drinking cycles collected via the rumen bolus.

### 2.2. Methodological Approach 1: Analysis of the Activity Index Recorded by Sensors (Rumen Bolus)

**Figure 1:**
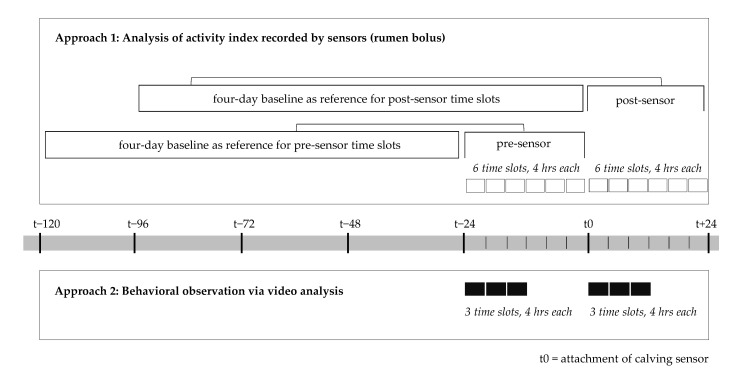
Schematic representation of the periods considered for analysis of behavior.

The first methodological approach was an analysis of the variability of activity [33,34,35,36,37,38]. All statistical calculations were performed using the software R [48] (packages: “tseries” [49], “car” [50], “DescTools” [51]). The aim of the statistical analysis was to determine whether the variability of the activity index recorded by the rumen bolus changed after attaching a calving sensor to the cow’s tail. In this process, the cows served as their own control [34,37]. By choosing a reference period of four days (see Figure 1), our study resembled the approach of Thorup et al. [34] who considered a reference period of two to eight days for analyzing variability of activity. To be representative of the actual activity, its variability was measured over a period of multiple days. At the same time, the exclusion of potential influences of cow drying off, being moved to the dry cow area, and being separated into a maternity pen was taken into consideration, resulting in a four-day reference period. Given the timeframe of five days prior to sensor attachment required for data analysis (see Figure 1) and at least 36 h between sensor attachment and calving, it was possible to include 18 cows in the analysis (five cows with Moocall sensors, 13 cows with CalveSense sensors). A period of 36 h was required between sensor attachment and calving to allow for a 24-h post-sensor study period while also accounting for natural pre-calving changes in behavior [4,52,53]. 

An analysis of the variability of activity was performed on the average activity index data across all 18 cows. We measured activity in six four-hour time slots, i.e., a total of 24 h consecutive to sensor attachment (t0 to t + 24; post-sensor) and compared it to the four-day baseline (t − 96 to t0) immediately prior to sensor attachment. To avoid any influences of the sensor attachment on the activity data of the first timeslot, we removed the data point(s) closest to the manually recorded time of attachment, thus generating a buffer of 10 to 15 min before the first data point was included in the analysis. To eliminate any bias from possible long-term trends, the activity data were detrended. The calculation of the first differences was sufficient to achieve stationarity for the time series before and after sensor attachment, as confirmed by the augmented Dickey–Fuller test (*p* < 0.01 for all tested cows and time series). All subsequent analyses were carried out on the detrended data. Since activity behavior may vary greatly from cow to cow [23,37], analyses of the detrended variability of the activity index were also performed separately for all cows. To assess whether activity behavior changed after attaching the calving sensor, we tested for homogeneity of variances between the four-day baseline and each of the post-sensor four-hour time slots, respectively (see Figure 1). Given the non-normal distribution of the data, we substituted the mean with the median, i.e., used the Brown–Forsythe test [54]. The term “variance” as such is technically not correct but is commonly used for reasons of comprehensibility [55,56]. Levene’s test and its modification, the Brown–Forsythe test, are frequently applied for testing the homogeneity of variances in different contexts [57,58,59,60,61,62,63,64].

The time at which the sensor is attached to the cow’s tail is defined as t0 and corresponds to a different time of day for each animal. To account for any possible influence of the diurnal activity pattern, we also conducted the Brown–Forsythe test for each of the six four-hour time slots on the day before the sensor was attached (pre-sensor). To remain consistent, the four days (t − 120 to t − 24) preceding this day were again used as a baseline (see Figure 1), resulting in a second baseline. This explains the required timeframe of five days prior to sensor attachment. In the process, the respective time slots, both pre- and post-sensor, refer to the same time of day for each animal (i.e., both first, second, …, and sixth time slots, respectively). The evaluation of 24-h pre- and post-sensor data enabled us to interpret changes in the variability of the activity index. More specifically, it allowed us to classify whether changes in the post-sensor activity behavior occurred more often than in the usual activity behavior of the cows (pre-sensor) and may thus be attributed to the calving sensor. To account for multiple testing, we applied the Bonferroni correction to maintain the significance level over all tests at 0.05 so that only those time slots for which *p* < 0.0083 were considered significant. 

The analysis of significances focused on the absolute number of significant time slots. If a sensor attached to the tail is perceived as disturbing and agitates the cow, she may try removing the sensor while suffering from restlessness. We therefore continued with the premise that time slots showing an increase in variability of activity with regard to the attachment of calving sensors were the relevant indicator. As cows exhibited periods of lower or higher variability of activity more frequently, for example, due to diurnal patterns of activity, we focused on changes in the number of time slots showing a significant increase in variability of activity in the post-sensor compared to the pre-sensor period. 

### 2.3. Methodological Approach 2: Behavioral Observation via Video Analysis

The second methodological approach was a conventional visual observation of the cows’ behavior recorded on video. The video recordings were available for nine of the 18 cows included in methodological approach 1 (cow ID 1 to 9 in Table A1). The subsample for video analysis included three cows with Moocall and six cows with CalveSense sensors. The assignment of animal behavior was performed on all nine cows by the same observer using the software Interact (Mangold International GmbH, Germany). All behavior patterns were observed for the first twelve hours consecutive to calving sensor attachment (t0 to t + 12) and for twelve hours at the same time of day the day before calving sensor attachment (t − 24 to t − 12) as the reference period. The twelve-hour periods were divided into three time slots of four hours each (see Figure 1). Again, the respective time slots, both pre- and post-sensor, referred to the same time of day for each cow (i.e., both first, second, and third time slots, respectively). In congruence with methodological approach 1, the cows thus served as their own control.

For the behavioral observation, an ethogram appropriate for the research question and based on published literature was developed. The ethogram included eight behavior patterns (see Table 1). 

Walking [13,25,65], standing [17,24,25,65], lying [17,24,25,26], and the number of standing and lying bouts [26,65,69,70] per time slot were captured visually. Furthermore, eating [17,18,24,25,66] and drinking [18,24,25] were included in the ethogram. Eating and drinking took place exclusively while standing, but both behaviors were analyzed and presented separately. To take this into account, eating and drinking were counted as “standing” when determining the number of lying and standing bouts.

Since a potential perception of calving sensors as disturbing depends on sensor position on the tail, we observed two further behaviors, tail raising [5] and rubbing the tail on objects, which may indicate that the cow was trying to change the position of the sensor on the tail. To differentiate between slight tail movements, tail raising was defined as lateral >90°. Tail-rubbing was performed on another cow in the maternity pen, on the penning, or on the water trough. In addition to the observation of individual animal behavior, social interaction [65,67,68] was recorded. 

Central tendencies were compared to evaluate whether changes in the analyzed behaviors occurred after attachment of calving sensors. Due to the non-normal distribution of the data, the Wilcoxon test for paired samples was applied. For each behavior, the medians of paired time slots were compared (i.e., first time slot pre- and post-sensor, second time slot pre- and post-sensor, and third time slot pre- and post-sensor, respectively). The sample of each time slot consisted of nine cows. Due to the sample size, a continuity correction was included in all Wilcoxon tests.

## 3. Results and Discussion

To interpret the results of our analyses, a brief overview of cows with relevant health documentation or showing abnormalities (Section 3.1) commences this section. Subsequently, the results on variability of activity and on behavioral observation (Section 3.2 and Section 3.3) are interpreted and discussed. 

### 3.1. Documentation Concerning Calving Sensor Attachment, Abnormalities after Calving Sensor Attachment, and Health

On the dairy research and demonstration farm, a total of 36 CalveSense devices were attached to the tail of cows, of which four devices (11.1%) fell off. Neither pressure marks nor swellings on the tail were documented after attachment of CalveSense devices. Of the total of 37 Moocall devices attached, 23 (62.2%) did not remain on the tail because they either fell off (and were then reattached) or were removed by the barn staff due to pressure marks, swellings, or technical problems (e.g., battery often empty although sensor charged). In eight of these 23 animals, pressure marks or swellings were documented during the twice daily routine inspection, whereupon the Moocall sensor was immediately removed. Due to the large number of Moocall sensors that did not remain on the tail, there were comparatively more cows with a CalveSense device in the analyzed sample of 18 animals.

Regarding the health documentation of the cows in the study, we focused on two weeks before and two weeks after attachment of the calving sensor. During this period, metaphylaxis against hypocalcemia was performed in eight of the 18 cows. However, it was carried out more than 24 h after the calving sensor was attached. Four cows (7, 12, 14, and 17) suffered from hypocalcemia after calving (diagnosed four, eight, two, and two days after attachment of the calving sensor, respectively). Also, after calving, one cow suffered from mastitis (cow 16; diagnosed four days after attachment of the calving sensor), one from metritis (cow 4; diagnosed ten days after attachment of the calving sensor), and one from retentio secundinarum (cow 3; diagnosed three days after attachment of the calving sensor). 

Based on the farm staff’s protocol, three of the 18 animals included in the analysis were identified to show abnormalities after attaching a calving sensor to their tail:

- In cow 2, conspicuous activity behavior was observed immediately after attaching the sensor (CalveSense) to the tail. She rubbed her tail heavily on the water trough for the first 15 min, although this decreased afterwards (also observed in video).
- About an hour after the calving sensor (Moocall) was attached to cow 7, the fit of the sensor on the tail had to be readjusted. The cow was fixed in the feed fence for a short time and the sensor was reattached (attachment: 08:24 a.m.; reattachment: 09:35 a.m.).
- Cow 15 showed discomfort in her activity behavior 23 h and 15 min after attachment of the calving sensor (Moocall). As pressure points and slight swelling were visible on the tail, the sensor was removed immediately (no video available).

### 3.2. Methodological Approach 1: Changes in the Variability of the Activity Index

On average across all 18 cows analyzed, the mean absolute deviation around the median (MAD) values as well as the medians were in a similar range for both of the four-day baselines (see Table 2). We could therefore assume that the selected baselines offered a stable representation of the usual activity behavior. This allowed for a comparison of each time slot with its respective baseline and subsequently the classification of deviations in activity behavior. The Brown–Forsythe test, applied to analyze variability of activity of all 18 cows, revealed that three of the pre-sensor and none of the post-sensor time slots showed a significant increase in the variability of activity. In addition, a significant decrease in the variability of activity was detected in one time slot pre-sensor, which was, however, outside of the focus of our investigation. In summary, the number of time slots showing a significant increase in the variability of the activity index in the post-sensor time slots was not increased compared to the pre-sensor time slots. On average across all 18 cows, an increase in the variability of the activity index after calving sensor attachment indicating severe agitation as a stress response as documented in other studies (e.g., [12,14,16,18,20]) was not observed.

**Figure 2:**
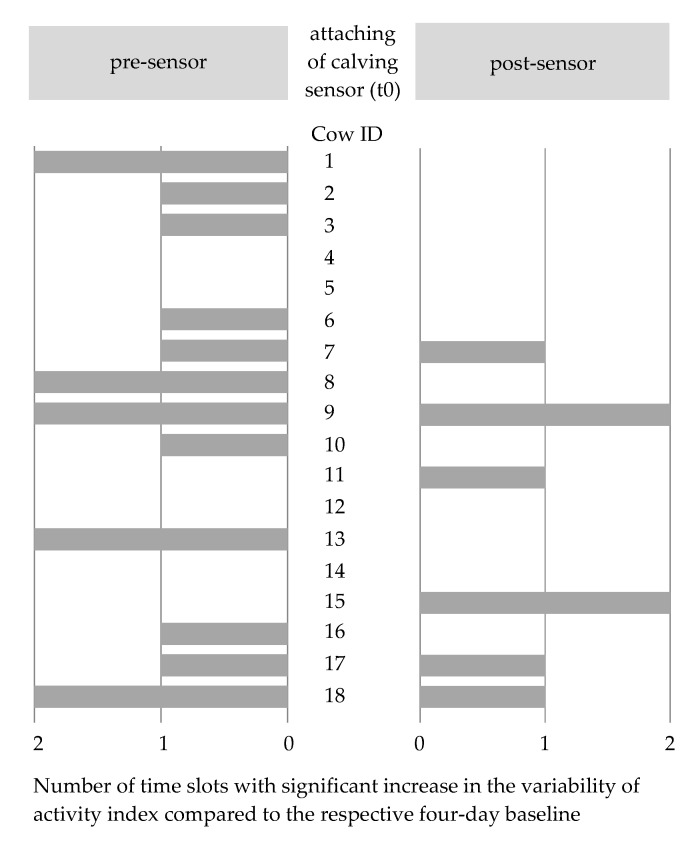
Depiction of the number of time slots pre-sensor (t − 24 to t0) and post-sensor (t0 to t + 24), showing a significant increase in the variability of the activity index using the respective four-day baseline as a reference (Brown–Forsythe test: *p* < 0.05, Bonferroni-corrected *p* < 0.0083).

In addition to the overall evaluation of all 18 cows shown in the section above, individual animal investigations elicited further information. Again, the analysis of individual cows revealed that, for both four-day baselines of any given cow, the MAD values as well as the medians were in a similar range (see Table A2). Figure 2 visualizes the absolute number of pre-sensor and post-sensor time slots showing a significant increase in the variability of activity relative to the respective four-day baseline (Brown–Forsythe test: *p* < 0.05). After the calving sensor was attached, none of the 18 cows exhibited a constant significant increase in the variability of activity in all six post-sensor time slots, while for twelve out of 18 cows no significant increase in the variability of activity could be detected in any of the post-sensor time slots. Four cows revealed a significant increase in the variability of activity in only one of the six post-sensor time slots and only two cows showed a significant increase in the variability of activity in two of the six post-sensor time slots. Considering the six cows that showed a significant increase in the variability of activity in one or two post-sensor time slots, a similar significance pattern in the pre-sensor time slots emerged for four of them. Cows 11 and 15, however, did not reveal a significant increase in the variability of activity in any of the pre-sensor time slots.

Cows 7 and 15, for which abnormalities were visually detected by the farm staff within 24 h of attaching the calving sensor, showed a significant increase in the variability of activity in the first time slot following the attachment of the calving sensor (t0 to t + 4), thus making them the only cows to show such immediate reactions (see Table A2). In cow 7 (readjustment of the sensor in time slot t0 to t + 4), the Brown–Forsythe test did not reveal statistically significant increases in the variability of activity occurring in the subsequent time slots (t + 4 to t + 24). Cow 15, however, showed a significant increase in the variability of activity also in the time slot t + 21 to t + 24 h post-sensor (see Table A2). In this period, tail swelling was detected, and the calving sensor was removed by the farm staff. 

Findings on cow behavior should be interpreted in a differentiated manner because reasons for changes in cow behavior may be rooted in a variety of causes. Changes in the activity of a cow can be attributed to individual animal behavior, diurnal patterns of activity, parity, stage of lactation, disease, estrus, and external effects [32,33,34,71,72], among other aspects. Behavioral changes may also be explained by a rebound effect, which has been demonstrated, for example, around the time of calving, with different surface types, or lying deprivation in dairy cattle [52,73,74]. As individual cow analyses revealed that some of the post-sensor time slots showing a significant increase in the variability of activity also showed statistical significance for the same time of day on the previous day, diurnal patterns of activity [32] became apparent.

Since a maximum of two of the six post-sensor time slots for each cow revealed a significant increase in the variability of activity, behavioral changes potentially caused by calving sensor attachment seem to have subsided after a short time. If the attachment of a calving sensor led to agitation in a cow, it can be assumed that this was only temporary. Several studies found that cows are able to acclimate to changes [13,18,75]. Concerns about the negative effects on animal welfare should therefore be greater in the presence of evidence of significant long-term changes in activity behavior. However, none of the 18 animals analyzed revealed significant changes in the variability of activity over the entire period of 24 h post-sensor attachment. In the two cows that did show a significant increase in the variability of activity in the first time slot post-sensor (cows 7 and 15), the significant result was not reproduced in the ensuing time slots. Farmers interviewed by Lind and Lindahl [10] reported that negative animal behavior lasted up to one hour after attaching a Moocall. Consequently, their observations are consistent with our results.

We applied the Brown–Forsythe test for comparing the variability of activity between the four-day baselines and four-hour time slots, i.e., between samples of different sizes. However, since observation of a sole 24-h time slot post-sensor would have been too imprecise, a subdivision into time slots was deemed appropriate. In the literature, it was reported that the Brown–Forsythe test is robust to unequal sample sizes [55,56]. 

The Bonferroni correction applied to account for Type I error accumulation in multiple testing is known to be conservative compared to alternative correction methods [76,77]. There is controversy about its use because reduction of the Type I error is accompanied by an increase in the risk of Type II error, leading to actual differences not being detected. However, as a low number of multiple tests was conducted in our evaluation, the Bonferroni-corrected significance level was not minimized to such an extent that the analysis would no longer have yielded any significant outcomes. A previous analysis of the significant time slots without Bonferroni correction revealed that the overall conclusion of methodological approach 1 remained the same, since Type I error accumulation affected both pre- and post-sensor time slots equally. This is a crucial point of our study, as our focus was on the change in the number of significant time slots between pre- and post-sensor periods rather than their absolute numbers.

The activity index used for methodological approach 1 is based on a proprietary algorithm that is not open source. Therefore, the detailed calculation of the activity index is not known. Although this is a limitation of our study, it does not weaken the proposed methodological approach of analyzing the variability of the activity index rather than activity itself to monitor dairy cows’ behavior. Furthermore, since all analyzed data from all animals and all time slots are based on the same algorithm, all steps in our analysis are subject to the same limitations so that the results of the comparisons are not biased. The lack of details on the calculation of the proprietary algorithm therefore does not impact the basic conclusions of our study.

### 3.3. Methodological Approach 2: Behavioral Observation via Video Analysis

#### 3.3.1. Standing, Walking, Lying, Eating, Drinking

**Figure A1:**
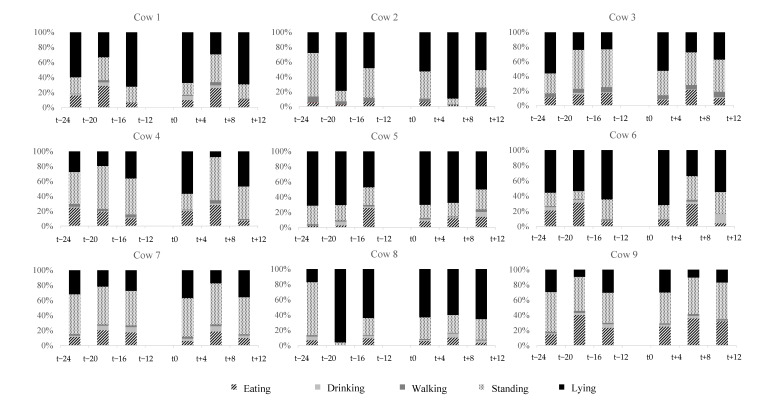
Relative share [%] of the behaviors eating, drinking, walking, standing, and lying of time slots analyzed.

The video analysis revealed that the behaviors standing, walking, lying, eating, and drinking were performed by all nine cows during the analyzed time. Behaviors being performed by a cow in the pre-sensor time slots were also observed in the post-sensor time slots (see Figure A1). Although different activity levels could be observed between the cows, the majority of post-sensor time slots revealed only slight changes in time spent being active compared to the previous day (see Figure A1). An exception was cow 8, which spent comparatively more time lying on the day of sensor attachment in the first time slot (t0 to t + 4), but comparatively less in the second time slot (t + 8 to t + 12). Considering all nine cows and their respective time slots, on average 45% (8 to 96%) were spent lying, 34% (4% to 69%) standing, 3% (0% to 8%) walking, 2% (0% to 12%) drinking, and 15% (0% to 40%) eating (see Figure A1).

**Figure 3:**
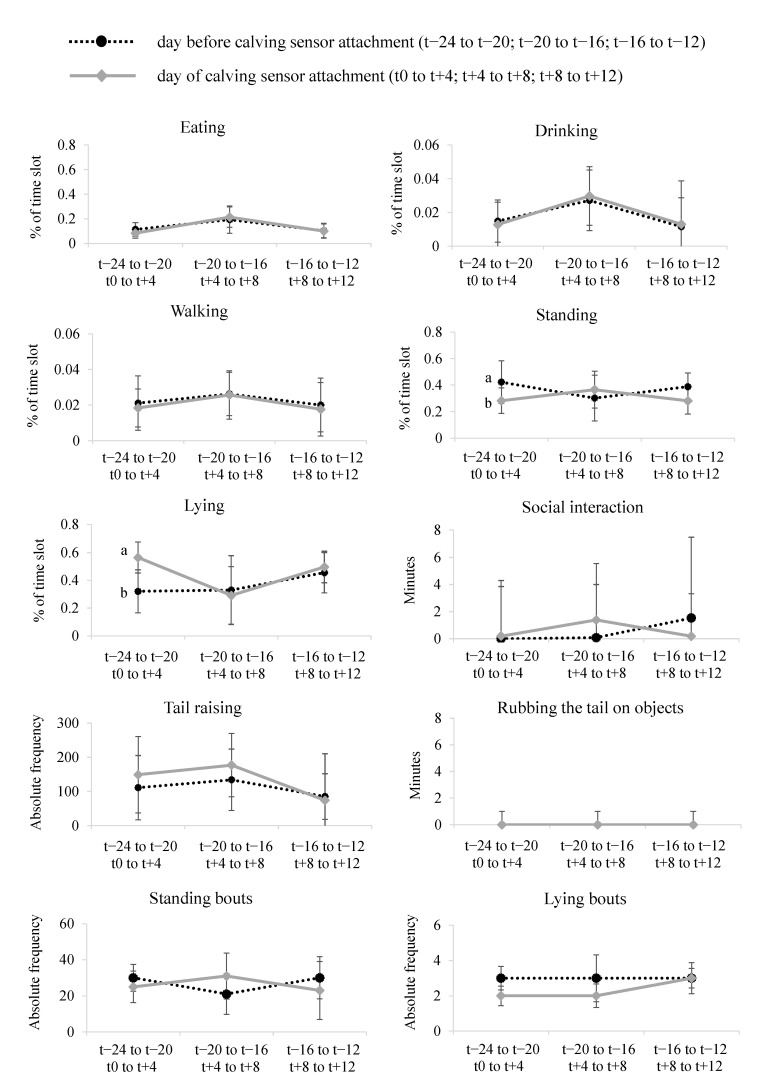
Medians of the behaviors observed by video analysis in the respective three time slots before (black dashed line) and after (gray solid line) calving sensor attachment (*n* = 9) (For each behavior, the black dashed lines represent the three pre-sensor time slots and the gray solid lines the three post-sensor time slots. Significant mean differences (*p* < 0.05) identified by the Wilcoxon test between the respective first, second, and third time slots pre- and post-sensor are marked with different superscripts (a, b). Mean absolute deviation around the median (MAD) is added).

The comparison of the behavior observed by means of video analysis in the pre- and post-sensor period is shown in Figure 3. Only the first time slots (t − 24 to t − 20; t0 to t + 4) of the behaviors standing and lying (*n* = 9) differed significantly in their means. Considering these time slots, the cows spent comparatively less time standing and more time lying on average after calving sensor attachment. However, these mean differences were mainly due to activity changes of cow 8 (see Figure A1). When excluding cow 8 from the Wilcoxon test, the sample of the remaining eight animals did not show any significant differences in the mean values of the behaviors standing and lying (*p* > 0.05). 

As lying and standing behavior are used as a sign of well-being in cattle, they have been assessed in cow behavior studies to answer a variety of research questions. These studies investigated changes in standing and lying subject to a variety of influences such as management factors, stall size and configuration, stocking density, heat stress, social ranking, overall health status, and pen layout and flooring [31,69]. The type of flooring may have a substantial impact on standing and lying, as cows reduce their number of standing and lying bouts on floorings considered uncomfortable, indicating an avoidance of frequent changes of position from lying to standing [70]. However, an increased number of standing and lying bouts as well as higher stepping rates, more frequent step and kicking behavior, and higher activity levels were recorded as signs of restlessness and stress responses in cows [12,16,17,18,20]. As we did not find any restrictions in the basic activity behaviors standing, walking, lying, eating, and drinking in eight of the nine cows observed in methodological approach 2, we cannot assume a general discontent of the cows. Only cow 8 changed especially its lying and standing behavior, as she spent more time lying and less time standing in the first time slot post-sensor compared to pre-sensor. However, this may be interpreted as a mere shift of lying and standing time between the first two pre- and post-sensor time slots, respectively (e.g., rebound effect).

#### 3.3.2. Tail Raising and Rubbing the Tail on Objects

Considering tail raising and rubbing the tail on objects, the Wilcoxon test did not show any significant mean differences between the respective pre- and post-sensor time slots (see Figure 3). However, an individual analysis of the cows provided further insights: Cows 2, 7, and 8 responded to the calving sensor attachment with an increased frequency of tail raising. In the post-sensor time slots, they performed the behavior of tail raising on average 2.0, 2.8, and 5.2 times as often (first, second, and third time slot) as in the pre-sensor time slots. While the analysis of activity index variability (methodological approach 1) did not show any significant results for the post-sensor period for cows 2 and 8, it revealed a significant increase in the variability of the activity index for the first post-sensor time slot (t0 to t + 4) for cow 7. Thus, the required readjustment of the sensor on the tail by farm staff during this time slot resulted in both an increased frequency of tail raising and an increase in the variability of the activity index in cow 7.

Whereas tail raising was performed by all nine cows, rubbing the tail on objects was observed only in cows 2 and 8. Both did not rub their tails on objects in the pre-sensor time slots, but in the post-sensor time slots for 3.1 (cow 2) and 0.8 (cow 8) minutes on average per time slot. For cow 2, rubbing of the tail on objects was predominantly observed in the first post-sensor time slot and for cow 8 in the first and second post-sensor time slots. Presumably, a certain degree of adaption can also be interpreted for the behavior of rubbing the tail on objects, reinforcing the findings of the Lind and Lindahl study [10], in which negative animal behavior reported by farmers persisted for up to one hour after calving sensor attachment, and of the Giaretta et al. [5] study, in which increased eating behavior after sensor attachment was characterized as temporary. Individual animal behavioral investigations via video observation leads to the assumption that especially tail raising (see [5]) and rubbing the tail on objects were appropriate, sensitive parameters to recognize that individual cows found the calving sensor uncomfortable and tried to remove it. However, this is not a reaction that occurred in all cows. The extent to which an increase in tail raising and rubbing the tail on objects can be considered stress in the cow is still an open question and would require more data for validation. 

#### 3.3.3. Social Interaction

Social interaction was observed in all nine cows analyzed. The observed behaviors predominantly included sniffing head, sniffing body, and social licking, and are therefore described as non-agonistic social interaction [67]. Sniffing head or body was observed in all nine cows. Only one gentle pushing (cow 8; time slot t − 16 to t − 12) and one head butting (cow 5; time slot t + 8 to t + 12) were observed as agonistic social interactions. Compared to the pre-sensor time slots, less social interaction was observed in the post-sensor time slots in five cows (1, 4, 6, 7, 9) and more social interaction was observed in four cows (2, 3, 5, 8). However, Wilcoxon testing did not indicate any significant mean differences between the respective compared pre- and post-sensor time slots (see Figure 3).

Social behavior [65,67,68] is an important welfare issue as stable social relationships in a dairy herd can help to reduce the effects of stressful conditions on animals. However, herding cows may also lead to aggression or social disturbance. For example, inappropriate housing conditions can cause social stress and aggressive behavior [68]. Since there were only two cows in the maternity pen simultaneously in our research facility, the results of social interaction should be interpreted carefully, as social interaction is usually recorded in loosely housed, larger dairy herds (e.g., [67]). Winckler et al. [78] already noted that the validity of analyzing social interaction over a short period of time may be limited due to high inter-day variation. It is known that the social interaction of licking specifically is perceived as comforting by animals [79]. For example, Galindo and Broom [79] compared social interaction of lame and non-lame cows. In lame cows, more non-agonistic social interaction, including licking, was observed on average. It was concluded that licking has a role in alleviating discomfort by looking for comfort from other cows. Although no statistically significant mean differences in social interaction were found in our study, it is striking to note that cow 8—which also exhibited an increased frequency of tail raising and rubbing the tail on objects in the post-sensor time slots—showed the greatest post-sensor increase in social interaction, thus possibly coping with discomfort.

### 3.4. General Discussion

Similar to what was described by Giaretta et al. [5], attaching a sensor to the tail did not generally alter the ethological pattern of the animals we analyzed. In Giaretta et al. [5] as well as in our study, no difference in tail movement after calving sensor attachment was detected across animals. Nevertheless, compared to Giaretta et al. [5], we analyzed tail raising continuously and over a longer period of time, finding an increase in the frequency of tail raising in three of nine animals, which was mainly observed in phases, indicating that tail raising should be recorded without gaps.

As also reported in Lind and Lindahl [10] and Voss [8], we point out that attaching a sensor with a ratchet (as is the case with the Moocall sensor) to an animal’s tail is challenging, even after long-time practice. The two studies reported swellings to the tail in 17% [8] and 87% [10] of animals to which a Moocall sensor was attached, and frequent dropping off the tail. Both these problems were also experienced on the dairy research and demonstration farm. In contrast, the fixation of the CalveSense device with an adhesive and tape did not cause any swellings to the animals’ tails. We thus reinforce that swelling of the tail due to calving sensor fixation is unacceptable (see [8,10]).

Applying two methodological approaches allowed for a multi-sided evaluation of the effect of attaching calving sensors on the behavior of cows. An analysis of the variability of the activity index provides additional insights to conventional visual observation of the active time of a cow (e.g., walking, eating). It was evident in many of the time slots that the variability of activity significantly increased while the median simultaneously decreased. This confirmed that an increase in the absolute activity level does not necessarily lead to an increase in the variability around this activity level (and vice versa). As analyzing the variability of general activity or specific movements is also a common methodological approach to predict diseases in cows [34,35,36], it may provide valuable information for describing the behavior of cows in further research and should receive more attention in animal behavioral research.

Both objective and subjective methods make an essential contribution to knowledge generation in animal behavior research [80]. While validity can be questioned for both subjective and objective methods, subjective methods are more prone to yielding different results when repeating an analysis of the same data set [80]. The reliability of animal observations can vary between several different observers (inter-observer reliability) as well as between observations repeated by one person (intra-observer reliability) [80,81]. The analysis of sensor-recorded activity data with regard to the variability of activity represents an objective and time-efficient solution that provides valuable information for the evaluation of animal behavior. 

Stress situations usually trigger a reaction that is a combination of both physiological and behavioral parameters [2,11,28]. In addition to behavioral indicators, physiological parameters such as hormone measurements, heart rate, or respiration (e.g., [15,16,29]) would have provided a further gain in information, and potentially would have reacted more quickly or more sensitively to the attachment of sensors to the cows’ tails. Additionally, some of the behaviors investigated in our study are described as maintenance behavior (e.g., lying, walking, intake of food and water [5]). Since animals are highly motivated to perform them, these behaviors are characterized by high resilience and therefore may not always be sensitive indicators for capturing animal responses [80]. Related to this, cows sometimes remain calm for very long periods of time despite discomfort or even pain [80,82], which makes the early detection of animal welfare problems challenging. Our results thus contribute to initial steps for identifying appropriate, sensitive behaviors and thereby answering the research question of whether the attachment of calving sensors leads to behavioral changes that could indicate disturbance by the sensors.

## 4. Conclusions

Based on the two methodological approaches, analysis of activity index and behavioral observation via video analysis, it can be concluded that there is little evidence that the attachment of calving sensors to the tails of dairy cows generally led to significant changes in behavior. No significant behavioral changes were found on average for the variability of the activity index and most visually analyzed behaviors, namely walking, eating, drinking, social interaction, tail raising, rubbing the tail, and the number of standing and lying bouts. On average across all cows analyzed, an increased lying time and reduced standing time was found in the first hours after calving sensor attachment, which, however, was sourced to one single cow and may be interpreted as a shift of lying and standing time. However, both methodological approaches revealed some abnormalities in individual cows. Individual cow investigations showed an increased number of time slots showing a significant increase in the variability of the activity index in two of 18 animals analyzed. Additionally, the two indicators, rubbing the tail on objects and tail raising, showed a temporarily increased occurrence after sensor attachment in two and three of nine cows analyzed, respectively. Since these two indicators may be interpreted as cow discomfort, further analysis is required to support the evidence. When calving sensors are attached to cows’ tails, short adaptation periods may occur in the animals, which, however, should be weighed against positive effects of calving prediction in terms of calf and cow welfare. However, the application of calving sensors must be limited to those devices that do not cause swelling or even injury to the tail.

## Figures and Tables

**Table 1:** Ethogram including the respective descriptions of the behaviors.

Unit	Behavior	Description
Duration of	Walking	The cow is moving all four legs (walking or running) [13]
	Standing	The cow is standing without moving [13,25]
	Lying	The cow is lying in different natural lying positions [65]
	Eating	The cow places its head above the feeding table and searches, masticates or sorts the feed (silage) [66]
	Drinking	The cow places its head over the water trough [24]
Frequency of	Tail raising ^1^	Lateral > 90°
Duration of	Rubbing the tail on objects ^1^	Rubbing the tail on objects (other cow in maternity pen, penning, or water trough)
	Social interaction ^1^	social licking: licking another cow’s head, neck, and/or shoulder areas sniffing head: head or muzzle stretched towards/maybe touching another cow’s head sniffing body: head or muzzle stretched towards/maybe touching another cow’s body gentle pushing: hard push of body against body head butting: blow with the forehead directed at another cow fighting: head-to-head pushing, sometimes followed by head to neck pushing and manoeuvring for position ([67], based on work from [68])

^1^ Observed parallel to the behaviors walking, standing, lying, eating, and drinking.

**Table 2:** Median and mean absolute deviation around the median (MAD) of the activity index, and results of the Brown–Forsythe test for the pre-sensor and post-sensor time slots using the respective four-day baseline as reference (*n* = 18).

	Baseline ^a^ (Pre-Sensor)	Baseline ^b^ (Post-Sensor)	t − 24 to t − 20	t − 20 to t − 16	t − 16 to t − 12	t − 12 to t − 8	t − 8 to t − 4	t − 4 to t0	t0 to t + 4	t + 4 to t + 8	t + 8 to t + 12	t + 12 to t + 16	t + 16 to t + 20	t + 20 to t + 24
MAD	0.16	0.17	0.23	0.25	0.19	0.16	0.09	0.30	0.24	0.15	0.12	0.11	0.13	0.23
median	−0.02	−0.02	−0.05	−0.06	−0.06	0.09	0.01	0.01	0.01	−0.05	0.01	0.01	−0.04	0.05
Brown–Forsythe test			sig.^I^	sig.^I^			sig.^D^	sig.^I^						

^a^ Four-day baseline (t − 120 to t − 24) as reference for pre-sensor time slots. ^b^ Four-day baseline (t − 96 to t0) as reference for post-sensor time slots. sig.^I^ significant increase in variability of activity, *p* < 0.05, Bonferroni-corrected *p* < 0.0083. sig.^D^ significant decrease in variability of activity, *p* < 0.05, Bonferroni-corrected *p* < 0.0083. t0 = attachment of calving sensor.

## Data Availability

The data presented in this study are available on request from the corresponding author. Restrictions apply to the availability of video data that show farm staff.

## Appendix A

**Table A1:** Breed, age, parity, and type of calving sensor attached of the 18 animals included in the analysis.

Cow ID	Breed	Age [years]	Parity	Type of Sensor	Included in Methodological Approach
1	Simmental	4.1	3	Moocall	1 ^a^ and 2 ^b^
2	Simmental	2.3	1	CalveSense	1 and 2
3	Brown-Swiss	2.1	1	CalveSense	1 and 2
4	Simmental	2.4	1	CalveSense	1 and 2
5	Simmental	10.4	8	CalveSense	1 and 2
6	Holstein	9.3	8	CalveSense	1 and 2
7	Simmental	8.6	7	Moocall	1 and 2
8	Simmental	4.3	3	Moocall	1 and 2
9	Brown-Swiss	5.5	4	CalveSense	1 and 2
10	Brown-Swiss	4.2	3	CalveSense	1
11	Simmental	4.3	3	CalveSense	1
12	Simmental	10.1	9	CalveSense	1
13	Holstein	6.6	5	Moocall	1
14	Simmental	6.8	5	CalveSense	1
15	Simmental	5.1	4	Moocall	1
16	Simmental	4.6	3	CalveSense	1
17	Holstein	4.2	3	CalveSense	1
18	Brown-Swiss	4.6	3	CalveSense	1

^a^ Analysis of the activity index recorded by rumen bolus. ^b^ Behavioral observation via video analysis.

**Table A2:** Mean absolute deviation around the median (MAD), median, and results of the Brown–Forsythe test for the pre-sensor and post-sensor time slots (four hours each) using the respective four-day baseline as reference.

Cow ID	Breed	Time Sensor Was Attached	Type of Sensor	Item	Baseline ^b^ (Pre-Sensor)	Baseline ^c^ (Post-Sensor)	t − 24 to t − 20	t − 20 to t − 16	t − 16 to t − 12	t − 12 to t − 8	t − 8 to t − 4	t − 4 to t0	t0 to t + 4	t + 4 to t + 8	t + 8 to t + 12	t + 12 to t + 16	t + 16 to t + 20	t + 20 to t + 24
1	S	09:15	M	MAD	0.66	0.73	1.10	0.69	0.43	0.98	0.62	0.55	0.44	0.60	0.53	0.26	0.76	0.33
				median	−0.01	0.01	0.59	−0.09	−0.37	0.06	−0.05	0.25	0.12	−0.10	−0.05	0.04	−0.07	0.12
				BF ^a^			sig.^I^			sig.^I^						sig.^D^		sig.^D^
2	S	19:20	C	MAD	0.66	0.65	1.00	0.50	0.52	0.60	0.73	0.59	0.86	0.50	0.67	0.55	0.48	0.29
				median	0.02	0.01	−0.37	−0.21	0.18	0.13	0.11	−0.09	−0.19	0.12	0.35	0.28	−0.04	−0.03
				BF			sig.^I^											sig.^D^
3	BS	09:50	C	MAD	0.79	0.77	0.97	1.28	0.51	0.55	0.42	0.58	0.97	0.90	0.70	0.63	0.55	0.60
				median	0.03	−0.03	−0.10	0.39	−0.27	−0.34	−0.04	0.06	0.03	0.15	−0.20	0.12	−0.21	0.23
				BF				sig.^I^			sig.^D^							
4	S	08:50	C	MAD	0.62	0.61	0.68	0.68	0.47	0.46	0.81	0.50	0.56	0.77	0.70	0.51	0.58	0.45
				median	0.03	−0.01	−0.10	0.30	−0.24	−0.20	0.30	−0.46	0.17	0.20	−0.38	0.12	−0.22	−0.02
				BF														
5	S	08:20	C	MAD	0.54	0.54	0.36	0.52	0.38	0.33	0.44	0.55	0.51	0.49	0.75	0.32	0.42	0.38
				median	−0.04	−0.02	0.01	−0.13	0.12	−0.04	−0.02	−0.01	−0.11	0.06	0.24	−0.05	0.10	−0.05
				BF														
6	H	08:50	C	MAD	0.36	0.35	0.28	0.54	0.27	0.45	0.12	0.35	0.21	0.37	0.29	0.27	0.34	0.19
				median	−0.03	−0.03	0.24	−0.16	−0.06	0.09	−0.07	0.09	0.09	0.09	−0.20	−0.19	−0.03	−0.04
				BF				sig.^I^			sig.^D^							sig.^D^
7	S	08:24	M	MAD	0.53	0.55	0.39	0.31	0.32	0.41	0.55	0.94	0.87	0.66	0.36	0.48	0.69	0.50
				median	0.00	0.01	0.11	−0.24	0.13	−0.03	−0.02	0.05	−0.11	0.02	0.15	0.20	−0.20	0.10
				BF								sig.^I^	sig.^I^					
8	S	17:55	M	MAD	0.82	0.82	1.44	0.65	0.79	1.03	0.70	1.28	0.71	0.90	0.54	0.82	0.96	0.77
				median	−0.01	−0.01	−0.21	−0.02	−0.01	0.02	−0.38	−0.33	−0.31	−0.25	−0.23	0.09	0.03	−0.19
				BF			sig.^I^					sig.^I^						
9	BS	08:25	C	MAD	0.97	1.06	0.34	0.50	1.10	0.66	2.11	1.92	0.78	0.27	0.50	1.70	0.61	2.05
				median	0.01	0.03	0.17	−0.145	0.25	−0.09	0.56	−0.87	−0.20	0.04	−0.02	−0.29	−0.11	−0.69
				BF			sig.^D^				sig.^I^	sig.^I^		sig.^D^		sig.^I^		sig.^I^
10	BS	09:00	C	MAD	0.84	0.86	0.67	0.73	2.05	0.73	0.93	0.68	0.97	0.85	0.46	0.67	0.52	0.65
				median	0.01	−0.07	−0.08	−0.32	−0.46	−0.43	−0.30	0.05	−0.06	0.05	−0.24	0.00	0.45	−0.31
				BF					sig.^I^						sig.^D^			
11	S	18:45	C	MAD	0.52	0.49	0.34	0.38	0.31	0.50	0.71	0.59	0.39	0.46	0.46	0.48	0.63	0.73
				median	−0.04	−0.02	−0.18	0.06	0.20	−0.10	−0.16	−0.22	−0.05	0.11	−0.10	−0.10	0.28	−0.47
				BF														sig.^I^
12	S	18:30	C	MAD	0.41	0.41	0.49	0.25	0.30	0.36	0.21	0.48	0.33	0.45	0.24	0.47	0.28	0.34
				median	−0.01	0.00	−0.18	0.18	−0.16	0.16	0.01	−0.05	−0.13	−0.06	0.20	−0.12	0.31	−0.10
				BF							sig.^D^				sig.^D^			
13	H	17:45	M	MAD	0.35	0.37	0.59	0.55	0.29	0.33	0.31	0.42	0.41	0.26	0.50	0.29	0.33	0.29
				median	0.02	0.01	0.11	−0.14	0.04	−0.03	−0.09	0.15	−0.08	−0.07	−0.11	−0.04	−0.13	0.04
				BF			sig.^I^	sig.^I^										
14	S	10:00	C	MAD	0.38	0.38	0.21	0.46	0.41	0.27	0.30	0.23	0.34	0.28	0.27	0.50	0.32	0.34
				median	0.01	0.01	0.20	−0.01	0.11	−0.22	0.04	−0.10	0.01	0.12	−0.12	−0.18	0.03	0.12
				BF														
15	S	08:50	M	MAD	0.48	0.46	0.45	0.55	0.32	0.53	0.24	0.37	0.86	0.57	0.45	0.40	0.34	0.75
				median	0.01	0.01	0.00	−0.03	0.04	0.15	0.01	0.07	−0.27	0.17	0.14	−0.30	−0.09	0.09
				BF							sig.^D^		sig.^I^					sig.^I^
16	S	19:20	C	MAD	0.56	0.54	1.09	0.35	0.25	0.41	0.53	0.41	0.37	0.32	0.41	0.47	0.39	0.43
				median	0.00	0.00	−0.32	−0.11	0.08	0.10	−0.02	0.06	−0.03	−0.01	0.06	0.04	0.07	0.01
				BF			sig.^I^		sig.^D^									
17	H	10:15	C	MAD	0.56	0.60	0.38	0.69	0.86	0.68	0.75	0.51	0.49	0.39	1.07	0.55	0.43	0.70
				median	0.02	0.06	0.25	0.19	0.27	0.18	−0.28	0.39	0.07	−0.17	0.25	−0.31	0.07	0.20
				BF					sig.^I^						sig.^I^			
18	BS	17:45	C	MAD	0.81	0.87	1.63	0.47	0.82	1.02	1.04	1.28	0.86	0.51	0.73	1.08	1.19	1.77
				median	−0.04	−0.07	−0.5	0.25	0.12	0.01	−0.28	0.35	−0.39	−0.14	0.22	0.43	−0.69	−0.26
				BF			sig.^I^					sig.^I^						sig.^I^

S = Simmental; BS = Brown Swiss; H = Holstein; C = CalveSense; M = Moocall. ^a^ Brown–Forsythe test. ^b^ Four-day baseline (t − 120 to t − 24) as reference for pre-sensor time slots. ^c^ Four-day baseline (t − 96 to t0) as reference for post-sensor time slots. sig.^I^ significant increase in variability of activity, *p* < 0.05, Bonferroni-corrected *p* < 0.0083. sig.^D^ significant decrease in variability of activity, *p* < 0.05, Bonferroni-corrected *p* < 0.0083. t0 = attaching of calving sensor.
